# Does *Helicobacter pylori* have a role in the pathogenesis of otitis media with effusion, or is it a fallacy??

**DOI:** 10.1007/s00405-025-09277-0

**Published:** 2025-03-14

**Authors:** Ayse Yasemin Gunduz, Mahmut Tayyar Kalcioglu, Serdal Celik, Oguz Ari, Riza Durmaz

**Affiliations:** 1 Department of Otorhinolaryngology, Faculty of Medicine, Istanbul Medeniyet University, Goztepe Prof. Dr. Suleyman Yalcin City Hospital, Kadikoy, Istanbul, Türkiye; 2 Department of Otorhinolaryngology, Mardin Training and Research Hospital, Artuklu, Mardin, Türkiye; 3 Central Research Laboratory, Ankara Yildirim Beyazit University, Ankara, Türkiye; 4 Department of Clinical Microbiology, Faculty of Medicine, Ankara Yildirim Beyazit University, Ankara, Türkiye

**Keywords:** Adenoid, Clarithromycin, Helicobacter pylori, Otitis media with effusion, Real-time polymerase chain reaction

## Abstract

**Purpose:**

*Helicobacter pylori*, causing chronic systemic infection, may colonize in middle ear milieu and conduce to effusion collection. Many investigations on relationship between pathogenesis of otitis media with effusion (OME) and *Helicobacter pylori* yielded conflicting results. We investigated *Helicobacter pylori* presence in effusion and adenoid samples of children having OME and in middle ear and adenoid samples of children with healthy middle ears to elucidate its role on OME pathogenesis.

**Methods:**

This prospective case-control study included 300 patients aged 1–12 years. One-hundred effusion samples collected from 100 children undergoing ventilation tube insertion and adenoidectomy due to chronic OME and adenoid hypertrophy formed study group, and 100 adenoid samples collected from adenoids of these children formed Group-1. One-hundred healthy-looking middle ear irrigation solutions collected from 100 children undergoing cochlear implantation formed Group-2. One-hundred adenoid samples collected from 100 children having no effusion and only undergoing adenoidectomy formed Group-3. After DNA isolation of samples, *Helicobacter pylori* 16 S rRNA and 23 S rRNA gene for clarithromycin-resistance were investigated by real time-polymerase chain reaction (Rt-PCR).

**Results:**

The median age of 300 children was 5, and 179 were boys and 121 were girls. *Helicobacter pylori* was detected by Rt-PCR in none (%0) of the 400 samples (200 middle ear, 200 adenoid).

**Conclusion:**

In this largest sample-size study utilizing updated molecular methods to date, negative results indicate that *Helicobacter pylori* does not play role as an active pathogen in polymicrobiality of OME, and adenoids do not serve as a reservoir for *Helicobacter pylori* in this process.

**Supplementary Information:**

The online version contains supplementary material available at 10.1007/s00405-025-09277-0.

## Introduction

Otitis media with effusion (OME), a major cause of hearing loss in children, is an important public health problem. As the disease primarily occurs in the critical developmental period of children and insidiously gets chronic with fluctuating mild-to-moderate hearing loss, the auditory deprivation adversely affects the development of central auditory processing pathways resultantly leading to deprived understanding, behavioral problems, irritability and sleep disorders, impaired communication and social skills, disrupted language development along with adversely affected academic lives of the affected children [1–4]. It is therefore of paramount importance to identify OME at early stage, initiate the necessary treatment and maintain follow-up.

There are two main views currently accepted in pathophysiology of OME. The first one which has long been studied is the disruption of pressure equilibrium system, where middle ear mucosa, mastoid air cells, and Eustachian tube (ET) function in harmony in a normal healthy ear [5]. In children, ET which has not completed its anatomical and physiological maturation prepares the ground for development of OME, and therefore OME is observed more frequently in pediatric population than in adults [6]. Recent studies showed that impairment in the balance between the production and evacuation of middle ear gas and mucus alone is not sufficient to explain pathogenesis and bacterial biofilm theory gained acceptance [7, 8]. Current molecular diagnostic methods revealed that OME is a polymicrobial disease. While main pathogens of the upper respiratory tract, *Streptococcus pneumoniae*,* Haemophilus influenzae* and *Moraxella catarrhalis*, are among the most common bacteria found in OME; *Alloiococcus otitis*, indigenous bacteria of the middle ear, is the most abundant bacterium found in OME [8–12]. In disease pathogenesis, these bacteria are thought to form a biofilm layer in middle ear to survive for long under favorable environmental conditions. Besides the middle ear’s own microbiota with possible role in pathogenesis of OME, according to the pathogen reservoir hypothesis, the main source of pathogenetic bacteria is upper respiratory tract, particularly adenoid tissue around the Eustachian orifice [13]. Which bacteria in the middle ear are involved in pathogenesis of OME and their source are still a subject of ongoing research.

*Helicobacter pylori (H. pylori)* is a bacterium often acquired early in life developing chronic inflammation in the host that may last lifelong [14]. With its systemic involvement, this bacterium was also detected in various tissues along the upper aerodigestive tract in head-neck region [15–21]. Presence of *H. pylori* in tonsil and adenoid tissues of children at different rates suggested *H. pylori* might also be found in the middle ear. To this end, adenoid tissue may act as a reservoir and the bacteria may spread through the ET, or the bacteria may reach the middle ear by passing through the ET with gastroesophageal and laryngopharyngeal reflux (GER/LPR) [22]. It is thought that middle ear, with its low oxygen and high carbon dioxide levels and near-neutral pH, has similar milieu characteristics required for colonization of bacteria with the environment when it is settled in gastric mucosa [22–24]. In literature, presence of *H. pylori* was investigated in effusion samples of children diagnosed with OME and bacteria were found at rates between 0 and 70% [22, 25]. Since the inflammatory processes of *H. pylori*-related gastritis and OME are similar in terms of lymphocyte predominance and goblet cell metaplasia, it is thought *H. pylori* may play a role in pathophysiology rather than being silent in the middle ear.

Recent studies showed different subtypes of the bacterium are involved in the disease process in *H. pylori* infections. Due to utilization of clarithromycin as first-line treatment in *H. pylori* infections for years, clarithromycin-resistant *H. pylori* strains are detected in a significant portion of infections [26]. Therefore, resistant strains should now be considered in addition to wild-type bacteria in *H. pylori* infections. In literature, presence of *H. pylori* was investigated in a limited number of middle ear effusions and the role of this bacterium in development of OME has not been clearly demonstrated [27].

In this study, presence of *H. pylori* in both middle ear and adenoid samples of children with OME and healthy children without effusion as the control was investigated by using the most advanced molecular methods, real time-polymerase chain reaction (Rt-PCR); and possible effect of *H. pylori* in pathogenesis of effusion and whether adenoid tissue acts as a reservoir for middle ear in this process will be clarified.

## Materials and methods

### Ethical approval

The study protocol was approved by Clinical Research Ethics Committee of Istanbul Medeniyet University Goztepe Prof. Dr. Suleyman Yalcin City Hospital on 02.11.2022 (Decision number:2022/0631) and conducted in accordance with Helsinki Declaration.

### Patient selection

This prospective case-control study was conducted at the Otorhinolaryngology Department of Istanbul Medeniyet University Goztepe Prof. Dr. Suleyman Yalcin City Hospital, a tertiary referral center in Türkiye, between 15.11.2022-01.11.2023. Children aged from 2 months to 12 years were included according to the target age group of the clinical practice guideline for OME management (2016 update) by the American Academy of Otolaryngology-Head and Neck Surgery Foundation [28].

Children with unilateral/bilateral effusion image with otoscopy, Type B or Type C2 tympanogram [29], and air-bone gap > 20 dB on pure-tone audiometry were followed up for 3 months after mucolytic administration; and those with persistent middle ear effusion requiring ventilation tube (VT) insertion formed the study group. Meanwhile, children with Grade 4 adenoid hypertrophy (≥ 75% nasopharyngeal obstruction in the sagittal plane with flexible nasopharyngoscopy) and thus with an indication of adenoidectomy were included to form the nasopharyngeal specimen arm of the study.

The study group comprised effusion samples from one ear of 100 children operated for unilateral/bilateral chronic OME. Adenoid samples from these 100 children who underwent concurrent adenoidectomy formed the intra-individual control group (Group-1). The inter-individual control groups comprised middle ear samples from one ear of 100 children who underwent unilateral/bilateral cochlear implant surgery with healthy-looking middle ears (Group-2) and adenoid samples from 100 children who underwent adenoidectomy but did not have OME (Group-3). The middle ear arm of the control groups of the study was decided to be composed of children undergoing cochlear implantation surgery, where surgical access to the middle ear is provided, so that the use of healthy subjects would not pose an ethical problem.

It was ensured that the children included in the study had not received antibiotics for at least 2 weeks prior to operation and did not have an acute upper respiratory tract infection or acute otitis media during the procedure. Additionally, children who had previous otologic surgery, adenoidectomy, or tonsillotomy/tonsillectomy; genetic syndromes with craniofacial anomalies such as Down syndrome or anatomical problems such as cleft palate; history of chronic medical disease; or other causes of airway obstruction such as turbinate hypertrophy or nasal polyps were excluded from the study. Informed consent was obtained from the parents of children meeting these criteria before surgery.

### Sample collection

All surgeries were performed under general anesthesia and sterile conditions. Eppendorf tubes of 1.5 ml were used for storage of collected samples.

**Fig. 1:**
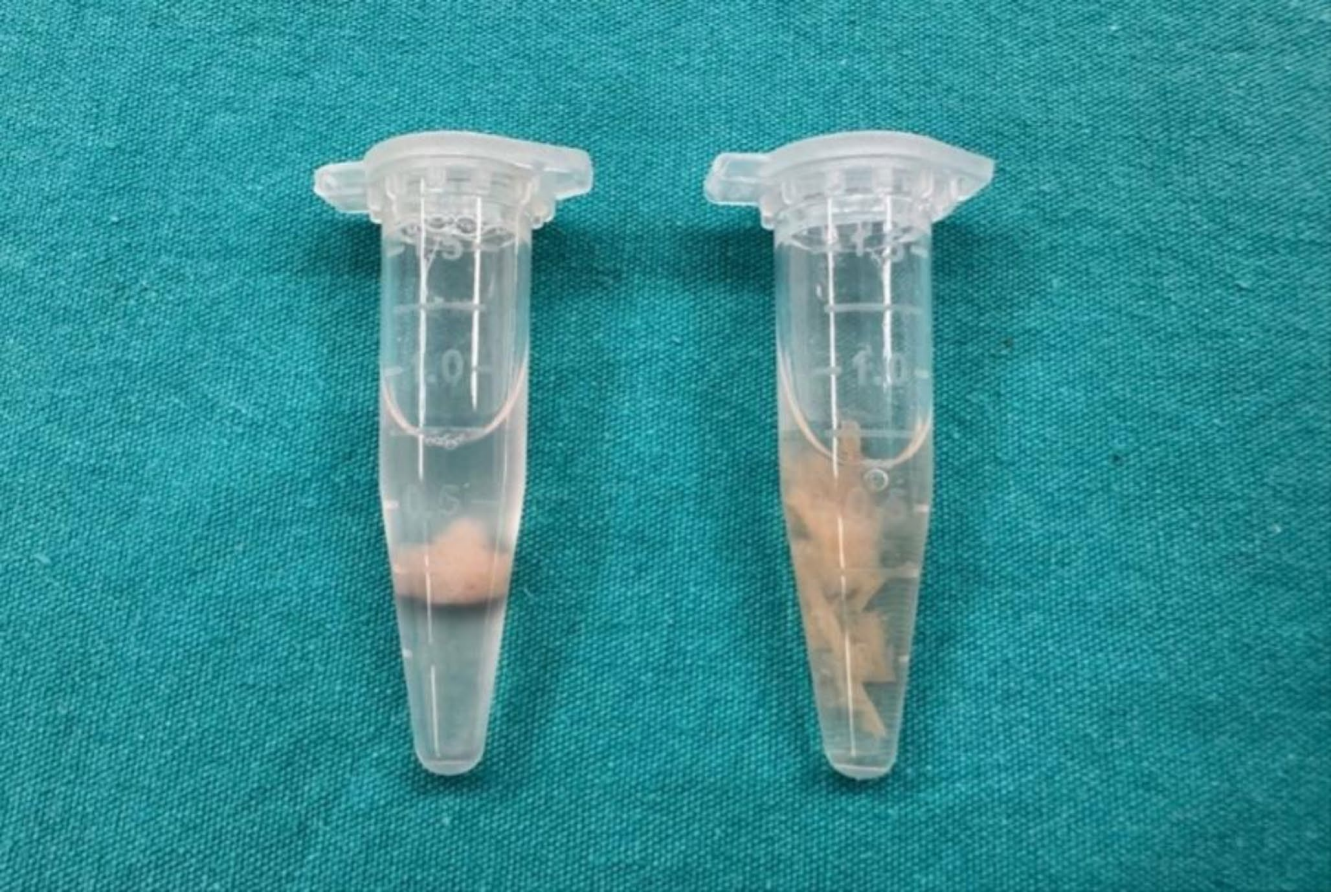
Adenoid tissue sample (left) and glue-form middle ear effusion sample (right) intraoperatively collected into Eppendorf tubes with saline

- **Middle ear effusion samples (study group)**: After irrigating external auditory canal with povidone iodine, middle ear effusion fluid sample in glue-form was collected using a microaspirator tip after paracentesis of tympanic membrane, and sterilely transferred from the microaspirator tip by irrigation with 1 ml saline into the Eppendorf tube (Fig. 1).

- **Adenoid samples (Group-1 and Group-3)**: After performing curettage adenoidectomies with adenotome, a 3 mm piece of the excised adenoid tissue was sectioned and sterilely transferred into the Eppendorf tube with 1 ml saline (Fig.  1).
- **Middle ear control samples (Group-2)**: After irrigating healthy middle ear cavities with saline during cochlear implantation surgery, the irrigation solutions were collected with 10 ml syringes and sterilely transferred to 1.5 ml Eppendorf tubes. Children found to have middle ear effusion or hypertrophic mucosa during surgery were excluded from Group-2.

### Storage and transport

Tubes with samples were placed in refrigerator at + 4 °C within 30 min for intermediate storage up to 3 h, and then transferred to deep-freezer at -32 °C for final storage until the samples were analyzed.

After collecting the desired number of samples, samples were transported with dry ice in a polystyrene box under cold-chain conditions to Ankara Diagen Biotechnological Systems Inc. research laboratory where experimental analyses would be performed.

### DNA isolation

DNA isolation from middle ear effusion and middle ear irrigation fluid samples was performed using DiaRex^®^ Blood Genomic DNA extraction kit (Catalogue No: BLD-5295, Diagen Inc., Ankara, Türkiye, www.diagen.com.tr). Briefly 200 µl of liquid sample was mixed with 25 µl proteinase K and 250 µl lysis buffer solution (LBD). After incubation at 56 °C for 15 min, 250 µl absolute ethanol was added and the entire lysate was transferred to a spin column. The column was centrifuged at 8000 g x 1 min. Then, after washing steps according to kit protocol, 100 µl elution solution was added and after incubation for 2 min, centrifugation at 8000 g x 1 min was performed, and genomic DNA was obtained.

DNA isolation from adenoid tissue samples was performed by using DiaRex^®^ Tissue Genomic DNA extraction kit (Catalogue No: TS-6826, Diagen Inc., Ankara, Türkiye, www.diagen.com.tr). Briefly, 250 µl LBD solution, 15 mg glass and 10 zirconia beads were added onto 20–30 mg tissue sample and milled at 4000 rpm 2 × 20 s. After homogenization, genomic DNA was isolated following the steps in kit’s protocol (https://www.diagen.com.tr/en/diarex-tissue-genomic-dna-extraction-kit).

### Investigation of *H. pylori* and clarithromycin resistance genes

Presence of *H. pylori* and clarithromycin resistance was investigated using DiaRD-HPykl Rt-PCR kit (Catalogue No: DiaRD-HPykl, Diagen Inc., Ankara, Türkiye, www.diagen.com.tr). DiaRD-HPykl Rt-PCR kit is a qualitative Rt-PCR assay designed to contain all necessary components to detect 16 S rRNA gene region of *H. pylori* DNA and A2142C/G and A2143G mutations in 23 S rRNA gene region responsible for clarithromycin resistance, resultantly emitting fluorescence in the presence of *H. pylori* specific DNA in sample (Table 1). As molecular Beacon probe targeted for clarithromycin resistance within the kit is designed to be wild-type specific, a signal is produced from the dye channel of this probe in the presence of clarithromycin-sensitive strain, whereas no signal is obtained in mutant resistant strain.

**Table 1:** DiaRD-HPykl Rt-PCR kit (DiaRD *H. pylori*-Clarithromycin resistance detection Rt-PCR kit). The kit contains an oligonucleotide mix which has primers to amplify *H. pylori* specific 16 S rRNA gene region and 23 S rRNA gene region of clarithromycin resistance in case of their presence in the sample; additionally, the kit includes primers and probe targeting beta-actin gene to detect possible PCR inhibitors. The qPCR master mix for the quantitative PCR analysis is the 2-times concentrated mixture containing the enzymes DNA polymerase, reverse transcriptase, electrolytes, buffer, enhancers, stabilizers, and fluorophore dyes, which are all necessary molecules for PCR cycle series. Positive control contains *H. pylori* DNA load of 1 × 10^2^ copies/µ and negative control contains sterile PCR grade water

Kit Contents			Tube count	Tube color
Oligonucleotide mix			1	Brown
qPCR master mix (2X)^a^			1	Blue
Positive control			1	Yellow
Negative control			1	White

PCR Mix Setup			Volume to be added	
Oligonucleotide mix			5 µL	
qPCR master mix (2X)^a^			10 µL	
Sample/Positive control/Negative control			5 µL	
**Final Volume**			**20 µL**	

PCR Cycle	Temperature	Time	Notes	
1	95^°^C	10 min	Initial denaturation	
50	95^°^C	15 s	Denaturation	
	62^°^C	60 s	Binding/Elongation^b^	

Dye channel		Gene regions		
HEX^c^		*H. pylori* 16 S rRNA gene		
FAM^d^		Clarithromycin		
CY5^e^		Actin beta gene		

^a^ qPCR master mix (2X): 2-times concentrated quantitative PCR master mix

^b^ HEX (Yellow Channel), FAM (Green Channel), CY5 (Red Channel) channels should be selected ON in order for the channels to collect the relevant dye and emit fluorescence signals in case of the presence and amplification of the target gene regions

^c^ HEX: Hexachlorofluorescein is a fluorescent dye giving emission in nanometer spectrum light of yellow. *H. pylori* 16 S rRNA region is labeled with HEX dye in this kit

^d^ FAM: Carboxyfluorescein is a fluorescent dye giving emission in nanometer spectrum light of green. Clarithromycin resistance gene region is labeled with FAM dye in this kit

^e^ CY5: Cyanine5 is a fluorescent dye giving emission in nanometer spectrum light of red. Actin beta gene region is labeled with CY5 dye in this kit. Actin beta gene is one of the most commonly used reference genes for controls because of its general expression and high stability in biologic specimens

### Statistical analysis

Descriptive statistics were expressed as number (n), percentage (%) and median (range, minimum value-maximum value). Test-retest correlation was presented with Pearson r. Kruskal-Wallis, ANOVA, Pearson Chi-square (χ2), and Fisher’s exact tests were planned to be used to compare *H. pylori* positivity and clarithromycin resistance rates between the study and control groups and to detect significant differences. Confidence interval (CI) was set at 95% and *p*-values < 0.05 were considered significant. Statistical procedures were planned to be performed with STATA 14.

## Results

A total of 300 consecutive pediatric patients fulfilling inclusion criteria within the study period were enrolled. The median age of these children was 5 years (range, 1–12), 179 (59.7%) were males and 121 (40.3%) were females. The median age of the 100 children concurrently undergoing VT insertion and adenoidectomy and forming the study group and Group-1 was 5 years (range, 1–10), 60 were boys and 40 were girls. The median age of 100 children in Group-2, consisting of 59 boys and 41 girls and undergoing cochlear implantation, was 3 years (range, 1–11); and the median age of 100 children in Group-3, consisting of 60 boys and 40 girls and undergoing adenoidectomy only, was 6 years (range, 3–12). The included ear sides of children whose middle ear samples were collected were randomly selected. Of the 100 effusion samples in the study group, 43 were from the right ear and 57 from the left ear; and of the 100 middle ear irrigation fluid samples in Group-2, 52 were from the right ear and 48 from the left ear.

**Fig. 2:**
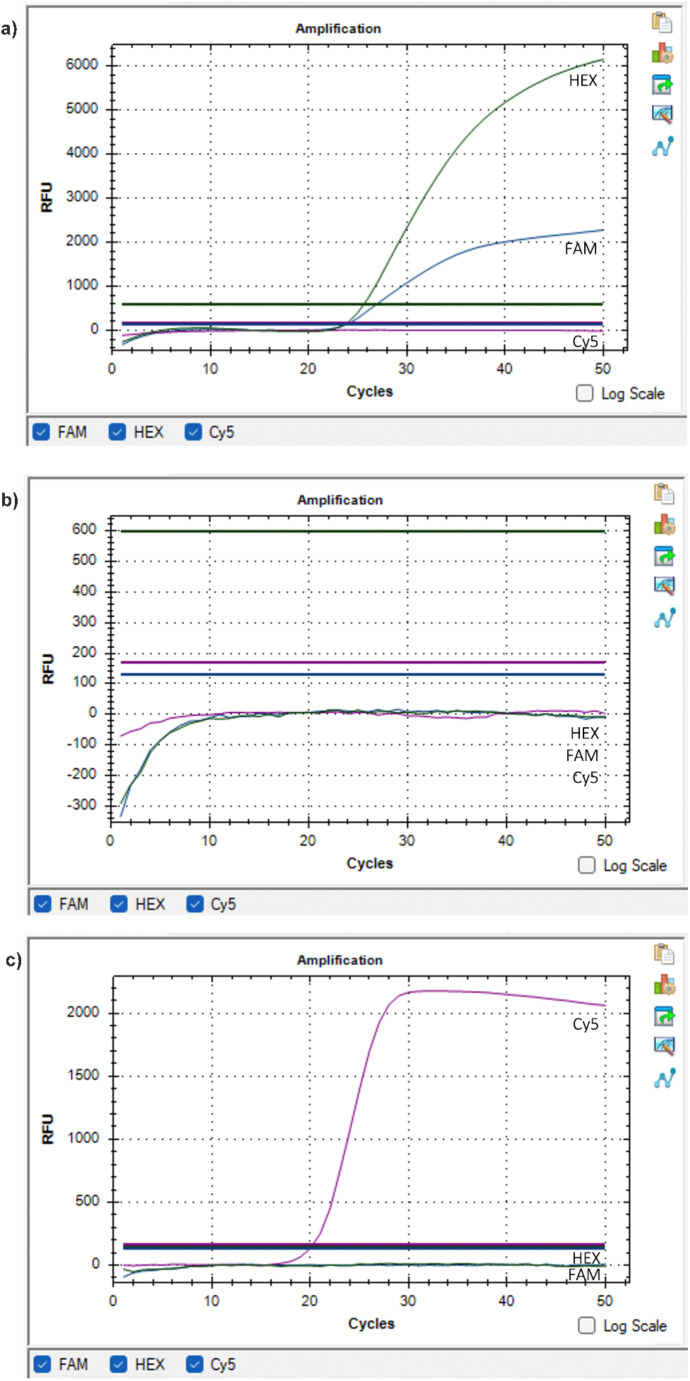
Fluorescence signal analysis result graphs of: (**a**) Positive control; (**b**) Negative control; (**c**) Clinical sample with negative test result. HEX (Hexachlorofluorescein), FAM (Carboxyfluorescein), and CY5 (Cyanine5) are fluorescent dyes giving emission in nanometer spectrum lights of yellow, green, and red, respectively. In this kit, *H. pylori* 16 S rRNA gene region is labeled with HEX dye, 23 S rRNA clarithromycin resistance gene region is labeled with FAM dye, and actin beta gene region is labeled with CY5 dye. As the positive control (**a**) contains clarithromycin sensitive *H. pylori* DNA, HEX and FAM dyes were released and fluoresced with amplification of both the 16 S rRNA and 23 S rRNA regions of *H. pylori* DNA. As the negative control (**b**) contains only sterile PCR grade water and no biologic material, none of the aforementioned genes were amplified, resulting in no fluorescence in any of these three dye channels. In our clinical samples (**c**), no fluorescence in HEX and FAM channels indicated the absence of *H. pylori* genes along with the presence of other cells in the samples with the amplification of actin beta gene used as the internal control. (RFU = Relative fluorescence units)

Rt-PCR studies were completed after DNA isolation for 400 samples in total consisting of the study group (*n* = 100), Group-1 (*n* = 100), Group-2 (*n* = 100), and Group-3 (*n* = 100); and *H. pylori* was not detected in any of the samples (0%) (see table in Online Resource, which lists the results of each sample). Result graphs demonstrating the fluorescence signal analysis of positive control, negative control, and clinical sample with negative test result are shown in Fig. 2. Test-retest method was used to maximize the reliability of results. For this, half of the samples were randomly selected and tested for the second time, and the same results were obtained with no *H. pylori* detected in any of the samples (0%) (test-retest correlation *r* = 1.00).

## Discussion

In this study, which is the first in literature to use the most specific methods in numerous diverse samples to elucidate the role of *H. pylori* in pathogenesis of OME, Rt-PCR analysis for *H. pylori* was negative in all middle ear fluid and adenoid tissue samples collected. This result achieved from 400 samples is very important for clarification of this issue, which is still controversial in literature. With these results, the argument that there are no *H. pylori* bacteria in middle ear fluid and adenoid tissue can now be justified.

In studies from literature investigating the presence of bacteria with different methods, *H. pylori* positivity in middle ear samples ranged from 0 to 70% [22, 25, 30–35], and similarly, it ranged from 0 to 64% in adenoid tissues [19, 22, 30, 31]. The fact of no detection or high levels of *H. pylori* positivity in middle ear effusions and adenoid tissues in these studies to date may be explained by methodological differences and limitations in most of these studies.

When literature is reviewed, studies investigating *H. pylori* included OME samples from a maximum of 132 patients and adenoid tissue samples from a maximum of 84 patients [31, 32]; that is, the number of patients and therefore the samples included are relatively low. This study, with a total of 400 samples (200 middle ear samples, 200 adenoid samples) from 300 pediatric patients, stands out as the largest sample size study to date investigating *H. pylori* in the middle ear and adenoid tissue, with a sample size similar to meta-analyses in literature [27, 36].

Some of the studies in literature were designed as cross-sectional studies and did not include control group [33, 34]. Even when some of the studies included a control group, age-matched control group was only found for adenoid tissue samples [35], and a control group was rarely found for middle ear samples, with a very limited sample size [37]. This study is the only study in literature including 3 different control groups together (intra-individual and inter-individual) with such a large sample size.

Various methods from routine histology-immunohistology to PCR have been used to detect *H. pylori* in biological samples [33, 35]; however, Rt-PCR offers the highest sensitivity and specificity by allowing the detection of even small numbers of microorganisms [27]. In our study, highly specific 16 S rRNA gene and 23 S rRNA gene showing clarithromycin resistance were analyzed together as target genes for detection of *H. pylori* and its clarithromycin resistance by Rt-PCR, a superior method to conventional PCR [38], and *H. pylori* was not detected in any of the samples. Studies in literature reporting *H. pylori* positivity in OME samples by 16 S rRNA-targeted PCR either found high rates of *H. pylori* infection in gastric lavage samples from patients with OME [37] or reported presence of reflux by clinical diagnostic questionnaire [33] or objective methods [39]. Interpreting these data, it is more reasonable to argue that, regardless of the pathogenesis of disease, significant GER/LPR may allow bacteria to reach adenoids and middle ear along with gastric contents [40].

There are many studies in literature supporting the argument that *H. pylori* does not play an active role in pathogenesis of OME. Jeyakumar et al. [25] and Khasawneh et al. [32] did not find *H. pylori* with PCR analyses in any of the effusion samples in the United States of America and Italy; and considering the positivity rates reported in other developing or endemic countries in literature, argued the bacterium has no role in development of OME in developed countries where *H. pylori* prevalence is low. Additionally, Khasawneh et al. [32] found GER in 1/3 of OME patients. In conclusion, as argued by Tasker et al. [41], they deduced there may be an association between OME and LPR, but *H. pylori* does not have a direct role in pathogenetic process of OME. Moreover, Sudhoff et al. [27] showed in their review the evidence is quite insufficient to say “OME may develop due to *H. pylori*”. In this context, it would not be erroneous to say even if *H. pylori* reaches the middle ear with LPR, it cannot remain active enough to affect middle ear physiology. Furthermore, slightly alkaline environment of middle ear of chronic OME patients, with a pH between 7.0 and 9.0, may not be the ideal environment for *H. pylori* to survive, contrary to popular belief. *H. pylori* is an acidophilic bacterium in presence of urea and can survive in environments with pH < 4 due to the urease enzyme it produces. In the absence of urea, it behaves as a neutrophilic organism and can survive in environments with pH values between 4.0 and 7.0 [42]. Various aquaglyceroporin channels that are expressed in the middle ear and ET, allow passage of other solutes including urea in addition to water [43]. As a consequence, it can be presumed that *H. pylori*, which will tend to behave as acidophile due to urea likely to be present in middle ear, will not be able to survive in slightly alkaline middle ear environment.

Numerous studies in literature investigated the hypothesis that adenoid tissue may serve as a reservoir for *H. pylori* during OME development, and findings have been discrepant from this hypothesis. These studies found either no or very low levels of *H. pylori* positivity in adenoid tissues [22]. In studies where *H. pylori* positivity was found, no significant difference was observed between OME patients and individuals with healthy middle ears [30]. Jelavic et al. [40] concluded in their review the bacterium can merely seed due to GER and be detected incidentally in nasopharynx and other head-neck regions, thus does not play a pathological role in otolaryngological diseases. In our study, no bacteria were detected in any effusion samples together with any adenoid samples, supporting the results of previous studies. Along with our results from such a large sample, it can now be more clearly said *H. pylori* lies outside the pathogenetic processes of development of OME or adenoid hypertrophy as a reservoir in OME.

## Conclusion

To effectively treat middle ear effusions, it is essential to understand pathogenetic process of the disease. In this study, performed on a large patient population utilizing the latest molecular techniques, *H. pylori* was not detected in any of the middle ear fluid and adenoid tissue samples, indicating this bacterium does not involve as a responsible pathogen in polymicrobial process of OME pathogenesis. Focusing on different etiological factors and agents in future studies on this topic will allow a more efficient and rational use of resources and provide results that can contribute to clinical practice.

## Electronic supplementary material

Below is the link to the electronic supplementary material.

Supplementary Material 1

## Data Availability

All data supporting the findings of this study are available within the paper and its Supplementary Information referred as Online Resource.
