# Supplementary material for: Does Helicobacter pylori have a role in the pathogenesis of otitis media with effusion, or is it a fallacy??
Source: Eur Arch Otorhinolaryngol. 2025 Mar 14;282(7):3561–9. doi: 10.1007/s00405-025-09277-0 (PMC12321910; doi:10.1007/s00405-025-09277-0)
Supplement: Supplementary file 1 — Supplementary Material 1 [file 405_2025_9277_MOESM1_ESM.pdf]

Supplementary Table. Results (N/A=Not applicable).

List No	Sample Type	Extraction Type	Elution Volume	DNA Extraction Kit Name	Catalog No	PCR Kit Name	Actin Beta CY5	Clarithromycin FAM	H. pylori HEX
**1**	Adenoid Tissue	DNA	100	DiaRex Tissue Kit	TS-6826	DiaRD-Hpykl	20,6	N/A	N/A
**2**	Adenoid Tissue	DNA	100	DiaRex Tissue Kit	TS-6826	DiaRD-Hpykl	18,2	N/A	N/A
**3**	Adenoid Tissue	DNA	100	DiaRex Tissue Kit	TS-6826	DiaRD-Hpykl	20,5	N/A	N/A
**4**	Adenoid Tissue	DNA	100	DiaRex Tissue Kit	TS-6826	DiaRD-Hpykl	20,2	N/A	N/A
**5**	Adenoid Tissue	DNA	100	DiaRex Tissue Kit	TS-6826	DiaRD-Hpykl	19,2	N/A	N/A
**6**	Adenoid Tissue	DNA	100	DiaRex Tissue Kit	TS-6826	DiaRD-Hpykl	22,5	N/A	N/A
**7**	Adenoid Tissue	DNA	100	DiaRex Tissue Kit	TS-6826	DiaRD-Hpykl	24,2	N/A	N/A
**8**	Adenoid Tissue	DNA	100	DiaRex Tissue Kit	TS-6826	DiaRD-Hpykl	23,1	N/A	N/A
**9**	Adenoid Tissue	DNA	100	DiaRex Tissue Kit	TS-6826	DiaRD-Hpykl	21,8	N/A	N/A
**10**	Adenoid Tissue	DNA	100	DiaRex Tissue Kit	TS-6826	DiaRD-Hpykl	22,1	N/A	N/A
**11**	Adenoid Tissue	DNA	100	DiaRex Tissue Kit	TS-6826	DiaRD-Hpykl	23,3	N/A	N/A
**12**	Adenoid Tissue	DNA	100	DiaRex Tissue Kit	TS-6826	DiaRD-Hpykl	21,9	N/A	N/A
**13**	Adenoid Tissue	DNA	100	DiaRex Tissue Kit	TS-6826	DiaRD-Hpykl	21,9	N/A	N/A
**14**	Adenoid Tissue	DNA	100	DiaRex Tissue Kit	TS-6826	DiaRD-Hpykl	21,6	N/A	N/A
**15**	Adenoid Tissue	DNA	100	DiaRex Tissue Kit	TS-6826	DiaRD-Hpykl	21,9	N/A	N/A
**16**	Adenoid Tissue	DNA	100	DiaRex Tissue Kit	TS-6826	DiaRD-Hpykl	22,0	N/A	N/A
**17**	Adenoid Tissue	DNA	100	DiaRex Tissue Kit	TS-6826	DiaRD-Hpykl	21,9	N/A	N/A
**18**	Adenoid Tissue	DNA	100	DiaRex Tissue Kit	TS-6826	DiaRD-Hpykl	24,8	N/A	N/A
**19**	Adenoid Tissue	DNA	100	DiaRex Tissue Kit	TS-6826	DiaRD-Hpykl	22,4	N/A	N/A
**20**	Adenoid Tissue	DNA	100	DiaRex Tissue Kit	TS-6826	DiaRD-Hpykl	25,2	N/A	N/A
**21**	Adenoid Tissue	DNA	100	DiaRex Tissue Kit	TS-6826	DiaRD-Hpykl	21,3	N/A	N/A
**22**	Adenoid Tissue	DNA	100	DiaRex Tissue Kit	TS-6826	DiaRD-Hpykl	22,2	N/A	N/A
**23**	Adenoid Tissue	DNA	100	DiaRex Tissue Kit	TS-6826	DiaRD-Hpykl	21,6	N/A	N/A
**24**	Adenoid Tissue	DNA	100	DiaRex Tissue Kit	TS-6826	DiaRD-Hpykl	22,1	N/A	N/A
**25**	Adenoid Tissue	DNA	100	DiaRex Tissue Kit	TS-6826	DiaRD-Hpykl	22,1	N/A	N/A
**26**	Adenoid Tissue	DNA	100	DiaRex Tissue Kit	TS-6826	DiaRD-Hpykl	31,7	N/A	N/A
**27**	Adenoid Tissue	DNA	100	DiaRex Tissue Kit	TS-6826	DiaRD-Hpykl	23,1	N/A	N/A
**28**	Adenoid Tissue	DNA	100	DiaRex Tissue Kit	TS-6826	DiaRD-Hpykl	24,5	N/A	N/A
**29**	Adenoid Tissue	DNA	100	DiaRex Tissue Kit	TS-6826	DiaRD-Hpykl	25,3	N/A	N/A
**30**	Adenoid Tissue	DNA	100	DiaRex Tissue Kit	TS-6826	DiaRD-Hpykl	24,0	N/A	N/A
**31**	Adenoid Tissue	DNA	100	DiaRex Tissue Kit	TS-6826	DiaRD-Hpykl	33,0	N/A	N/A

**32**	Adenoid Tissue	DNA	100	DiaRex Tissue Kit	TS-6826	DiaRD-Hpykl	22,2	N/A	N/A
**33**	Adenoid Tissue	DNA	100	DiaRex Tissue Kit	TS-6826	DiaRD-Hpykl	22,4	N/A	N/A
**34**	Adenoid Tissue	DNA	100	DiaRex Tissue Kit	TS-6826	DiaRD-Hpykl	22,0	N/A	N/A
**35**	Adenoid Tissue	DNA	100	DiaRex Tissue Kit	TS-6826	DiaRD-Hpykl	21,6	N/A	N/A
**36**	Adenoid Tissue	DNA	100	DiaRex Tissue Kit	TS-6826	DiaRD-Hpykl	22,7	N/A	N/A
**37**	Adenoid Tissue	DNA	100	DiaRex Tissue Kit	TS-6826	DiaRD-Hpykl	24,3	N/A	N/A
**38**	Adenoid Tissue	DNA	100	DiaRex Tissue Kit	TS-6826	DiaRD-Hpykl	21,8	N/A	N/A
**39**	Adenoid Tissue	DNA	100	DiaRex Tissue Kit	TS-6826	DiaRD-Hpykl	27,6	N/A	N/A
**40**	Adenoid Tissue	DNA	100	DiaRex Tissue Kit	TS-6826	DiaRD-Hpykl	21,4	N/A	N/A
**41**	Adenoid Tissue	DNA	100	DiaRex Tissue Kit	TS-6826	DiaRD-Hpykl	22,7	N/A	N/A
**42**	Adenoid Tissue	DNA	100	DiaRex Tissue Kit	TS-6826	DiaRD-Hpykl	22,4	N/A	N/A
**43**	Adenoid Tissue	DNA	100	DiaRex Tissue Kit	TS-6826	DiaRD-Hpykl	22,6	N/A	N/A
**44**	Adenoid Tissue	DNA	100	DiaRex Tissue Kit	TS-6826	DiaRD-Hpykl	23,5	N/A	N/A
**45**	Adenoid Tissue	DNA	100	DiaRex Tissue Kit	TS-6826	DiaRD-Hpykl	24,3	N/A	N/A
**46**	Adenoid Tissue	DNA	100	DiaRex Tissue Kit	TS-6826	DiaRD-Hpykl	21,8	N/A	N/A
**47**	Adenoid Tissue	DNA	100	DiaRex Tissue Kit	TS-6826	DiaRD-Hpykl	24,6	N/A	N/A
**48**	Adenoid Tissue	DNA	100	DiaRex Tissue Kit	TS-6826	DiaRD-Hpykl	21,8	N/A	N/A
**49**	Adenoid Tissue	DNA	100	DiaRex Tissue Kit	TS-6826	DiaRD-Hpykl	23,5	N/A	N/A
**50**	Adenoid Tissue	DNA	100	DiaRex Tissue Kit	TS-6826	DiaRD-Hpykl	21,5	N/A	N/A
**51**	Adenoid Tissue	DNA	100	DiaRex Tissue Kit	TS-6826	DiaRD-Hpykl	22,1	N/A	N/A
**52**	Adenoid Tissue	DNA	100	DiaRex Tissue Kit	TS-6826	DiaRD-Hpykl	21,8	N/A	N/A
**53**	Adenoid Tissue	DNA	100	DiaRex Tissue Kit	TS-6826	DiaRD-Hpykl	22,9	N/A	N/A
**54**	Adenoid Tissue	DNA	100	DiaRex Tissue Kit	TS-6826	DiaRD-Hpykl	22,1	N/A	N/A
**55**	Adenoid Tissue	DNA	100	DiaRex Tissue Kit	TS-6826	DiaRD-Hpykl	24,3	N/A	N/A
**56**	Adenoid Tissue	DNA	100	DiaRex Tissue Kit	TS-6826	DiaRD-Hpykl	20,8	N/A	N/A
**57**	Adenoid Tissue	DNA	100	DiaRex Tissue Kit	TS-6826	DiaRD-Hpykl	22,2	N/A	N/A
**58**	Adenoid Tissue	DNA	100	DiaRex Tissue Kit	TS-6826	DiaRD-Hpykl	23,9	N/A	N/A
**59**	Adenoid Tissue	DNA	100	DiaRex Tissue Kit	TS-6826	DiaRD-Hpykl	22,2	N/A	N/A
**60**	Adenoid Tissue	DNA	100	DiaRex Tissue Kit	TS-6826	DiaRD-Hpykl	21,5	N/A	N/A
**61**	Adenoid Tissue	DNA	100	DiaRex Tissue Kit	TS-6826	DiaRD-Hpykl	20,9	N/A	N/A
**62**	Adenoid Tissue	DNA	100	DiaRex Tissue Kit	TS-6826	DiaRD-Hpykl	22,6	N/A	N/A
**63**	Adenoid Tissue	DNA	100	DiaRex Tissue Kit	TS-6826	DiaRD-Hpykl	22,1	N/A	N/A
**64**	Adenoid Tissue	DNA	100	DiaRex Tissue Kit	TS-6826	DiaRD-Hpykl	23,8	N/A	N/A
**65**	Adenoid Tissue	DNA	100	DiaRex Tissue Kit	TS-6826	DiaRD-Hpykl	24,0	N/A	N/A

**66**	Adenoid Tissue	DNA	100	DiaRex Tissue Kit	TS-6826	DiaRD-Hpykl	22,8	N/A	N/A
**67**	Adenoid Tissue	DNA	100	DiaRex Tissue Kit	TS-6826	DiaRD-Hpykl	21,8	N/A	N/A
**68**	Adenoid Tissue	DNA	100	DiaRex Tissue Kit	TS-6826	DiaRD-Hpykl	35,8	N/A	N/A
**69**	Adenoid Tissue	DNA	100	DiaRex Tissue Kit	TS-6826	DiaRD-Hpykl	21,8	N/A	N/A
**70**	Adenoid Tissue	DNA	100	DiaRex Tissue Kit	TS-6826	DiaRD-Hpykl	22,4	N/A	N/A
**71**	Adenoid Tissue	DNA	100	DiaRex Tissue Kit	TS-6826	DiaRD-Hpykl	20,0	N/A	N/A
**72**	Adenoid Tissue	DNA	100	DiaRex Tissue Kit	TS-6826	DiaRD-Hpykl	21,6	N/A	N/A
**73**	Adenoid Tissue	DNA	100	DiaRex Tissue Kit	TS-6826	DiaRD-Hpykl	23,4	N/A	N/A
**74**	Adenoid Tissue	DNA	100	DiaRex Tissue Kit	TS-6826	DiaRD-Hpykl	22,0	N/A	N/A
**75**	Adenoid Tissue	DNA	100	DiaRex Tissue Kit	TS-6826	DiaRD-Hpykl	22,5	N/A	N/A
**76**	Adenoid Tissue	DNA	100	DiaRex Tissue Kit	TS-6826	DiaRD-Hpykl	22,2	N/A	N/A
**77**	Adenoid Tissue	DNA	100	DiaRex Tissue Kit	TS-6826	DiaRD-Hpykl	23,0	N/A	N/A
**78**	Adenoid Tissue	DNA	100	DiaRex Tissue Kit	TS-6826	DiaRD-Hpykl	21,5	N/A	N/A
**79**	Adenoid Tissue	DNA	100	DiaRex Tissue Kit	TS-6826	DiaRD-Hpykl	21,4	N/A	N/A
**80**	Adenoid Tissue	DNA	100	DiaRex Tissue Kit	TS-6826	DiaRD-Hpykl	23,3	N/A	N/A
**81**	Adenoid Tissue	DNA	100	DiaRex Tissue Kit	TS-6826	DiaRD-Hpykl	24,0	N/A	N/A
**82**	Adenoid Tissue	DNA	100	DiaRex Tissue Kit	TS-6826	DiaRD-Hpykl	22,0	N/A	N/A
**83**	Adenoid Tissue	DNA	100	DiaRex Tissue Kit	TS-6826	DiaRD-Hpykl	23,8	N/A	N/A
**84**	Adenoid Tissue	DNA	100	DiaRex Tissue Kit	TS-6826	DiaRD-Hpykl	25,2	N/A	N/A
**85**	Adenoid Tissue	DNA	100	DiaRex Tissue Kit	TS-6826	DiaRD-Hpykl	22,4	N/A	N/A
**86**	Adenoid Tissue	DNA	100	DiaRex Tissue Kit	TS-6826	DiaRD-Hpykl	22,2	N/A	N/A
**87**	Adenoid Tissue	DNA	100	DiaRex Tissue Kit	TS-6826	DiaRD-Hpykl	20,2	N/A	N/A
**88**	Adenoid Tissue	DNA	100	DiaRex Tissue Kit	TS-6826	DiaRD-Hpykl	22,3	N/A	N/A
**89**	Adenoid Tissue	DNA	100	DiaRex Tissue Kit	TS-6826	DiaRD-Hpykl	22,4	N/A	N/A
**90**	Adenoid Tissue	DNA	100	DiaRex Tissue Kit	TS-6826	DiaRD-Hpykl	22,5	N/A	N/A
**91**	Adenoid Tissue	DNA	100	DiaRex Tissue Kit	TS-6826	DiaRD-Hpykl	22,0	N/A	N/A
**92**	Adenoid Tissue	DNA	100	DiaRex Tissue Kit	TS-6826	DiaRD-Hpykl	22,0	N/A	N/A
**93**	Adenoid Tissue	DNA	100	DiaRex Tissue Kit	TS-6826	DiaRD-Hpykl	21,2	N/A	N/A
**94**	Adenoid Tissue	DNA	100	DiaRex Tissue Kit	TS-6826	DiaRD-Hpykl	23,5	N/A	N/A
**95**	Adenoid Tissue	DNA	100	DiaRex Tissue Kit	TS-6826	DiaRD-Hpykl	23,0	N/A	N/A
**96**	Adenoid Tissue	DNA	100	DiaRex Tissue Kit	TS-6826	DiaRD-Hpykl	22,6	N/A	N/A
**97**	Adenoid Tissue	DNA	100	DiaRex Tissue Kit	TS-6826	DiaRD-Hpykl	21,6	N/A	N/A
**98**	Adenoid Tissue	DNA	100	DiaRex Tissue Kit	TS-6826	DiaRD-Hpykl	21,8	N/A	N/A
**99**	Adenoid Tissue	DNA	100	DiaRex Tissue Kit	TS-6826	DiaRD-Hpykl	22,9	N/A	N/A

**100**	Adenoid Tissue	DNA	100	DiaRex Tissue Kit	TS-6826	DiaRD-Hpykl	24,9	N/A	N/A
**101**	Adenoid Tissue	DNA	100	DiaRex Tissue Kit	TS-6826	DiaRD-Hpykl	21,3	N/A	N/A
**102**	Adenoid Tissue	DNA	100	DiaRex Tissue Kit	TS-6826	DiaRD-Hpykl	22,1	N/A	N/A
**103**	Adenoid Tissue	DNA	100	DiaRex Tissue Kit	TS-6826	DiaRD-Hpykl	21,7	N/A	N/A
**104**	Adenoid Tissue	DNA	100	DiaRex Tissue Kit	TS-6826	DiaRD-Hpykl	23,5	N/A	N/A
**105**	Adenoid Tissue	DNA	100	DiaRex Tissue Kit	TS-6826	DiaRD-Hpykl	24,0	N/A	N/A
**106**	Adenoid Tissue	DNA	100	DiaRex Tissue Kit	TS-6826	DiaRD-Hpykl	21,1	N/A	N/A
**107**	Adenoid Tissue	DNA	100	DiaRex Tissue Kit	TS-6826	DiaRD-Hpykl	20,3	N/A	N/A
**108**	Adenoid Tissue	DNA	100	DiaRex Tissue Kit	TS-6826	DiaRD-Hpykl	21,2	N/A	N/A
**109**	Adenoid Tissue	DNA	100	DiaRex Tissue Kit	TS-6826	DiaRD-Hpykl	20,7	N/A	N/A
**110**	Adenoid Tissue	DNA	100	DiaRex Tissue Kit	TS-6826	DiaRD-Hpykl	21,7	N/A	N/A
**111**	Adenoid Tissue	DNA	100	DiaRex Tissue Kit	TS-6826	DiaRD-Hpykl	21,1	N/A	N/A
**112**	Adenoid Tissue	DNA	100	DiaRex Tissue Kit	TS-6826	DiaRD-Hpykl	25,1	N/A	N/A
**113**	Adenoid Tissue	DNA	100	DiaRex Tissue Kit	TS-6826	DiaRD-Hpykl	21,5	N/A	N/A
**114**	Adenoid Tissue	DNA	100	DiaRex Tissue Kit	TS-6826	DiaRD-Hpykl	32,4	N/A	N/A
**115**	Adenoid Tissue	DNA	100	DiaRex Tissue Kit	TS-6826	DiaRD-Hpykl	19,7	N/A	N/A
**116**	Adenoid Tissue	DNA	100	DiaRex Tissue Kit	TS-6826	DiaRD-Hpykl	21,0	N/A	N/A
**117**	Adenoid Tissue	DNA	100	DiaRex Tissue Kit	TS-6826	DiaRD-Hpykl	21,0	N/A	N/A
**118**	Adenoid Tissue	DNA	100	DiaRex Tissue Kit	TS-6826	DiaRD-Hpykl	23,1	N/A	N/A
**119**	Adenoid Tissue	DNA	100	DiaRex Tissue Kit	TS-6826	DiaRD-Hpykl	20,4	N/A	N/A
**120**	Adenoid Tissue	DNA	100	DiaRex Tissue Kit	TS-6826	DiaRD-Hpykl	19,7	N/A	N/A
**121**	Adenoid Tissue	DNA	100	DiaRex Tissue Kit	TS-6826	DiaRD-Hpykl	20,8	N/A	N/A
**122**	Adenoid Tissue	DNA	100	DiaRex Tissue Kit	TS-6826	DiaRD-Hpykl	20,8	N/A	N/A
**123**	Adenoid Tissue	DNA	100	DiaRex Tissue Kit	TS-6826	DiaRD-Hpykl	20,6	N/A	N/A
**124**	Adenoid Tissue	DNA	100	DiaRex Tissue Kit	TS-6826	DiaRD-Hpykl	20,1	N/A	N/A
**125**	Adenoid Tissue	DNA	100	DiaRex Tissue Kit	TS-6826	DiaRD-Hpykl	20,6	N/A	N/A
**126**	Adenoid Tissue	DNA	100	DiaRex Tissue Kit	TS-6826	DiaRD-Hpykl	22,0	N/A	N/A
**127**	Adenoid Tissue	DNA	100	DiaRex Tissue Kit	TS-6826	DiaRD-Hpykl	20,4	N/A	N/A
**128**	Adenoid Tissue	DNA	100	DiaRex Tissue Kit	TS-6826	DiaRD-Hpykl	20,6	N/A	N/A
**129**	Adenoid Tissue	DNA	100	DiaRex Tissue Kit	TS-6826	DiaRD-Hpykl	20,8	N/A	N/A
**130**	Adenoid Tissue	DNA	100	DiaRex Tissue Kit	TS-6826	DiaRD-Hpykl	22,3	N/A	N/A
**131**	Adenoid Tissue	DNA	100	DiaRex Tissue Kit	TS-6826	DiaRD-Hpykl	22,1	N/A	N/A
**132**	Adenoid Tissue	DNA	100	DiaRex Tissue Kit	TS-6826	DiaRD-Hpykl	20,9	N/A	N/A
**133**	Adenoid Tissue	DNA	100	DiaRex Tissue Kit	TS-6826	DiaRD-Hpykl	20,1	N/A	N/A

**134**	Adenoid Tissue	DNA	100	DiaRex Tissue Kit	TS-6826	DiaRD-Hpykl	20,9	N/A	N/A
**135**	Adenoid Tissue	DNA	100	DiaRex Tissue Kit	TS-6826	DiaRD-Hpykl	20,0	N/A	N/A
**136**	Adenoid Tissue	DNA	100	DiaRex Tissue Kit	TS-6826	DiaRD-Hpykl	23,2	N/A	N/A
**137**	Adenoid Tissue	DNA	100	DiaRex Tissue Kit	TS-6826	DiaRD-Hpykl	20,7	N/A	N/A
**138**	Adenoid Tissue	DNA	100	DiaRex Tissue Kit	TS-6826	DiaRD-Hpykl	23,0	N/A	N/A
**139**	Adenoid Tissue	DNA	100	DiaRex Tissue Kit	TS-6826	DiaRD-Hpykl	21,1	N/A	N/A
**140**	Adenoid Tissue	DNA	100	DiaRex Tissue Kit	TS-6826	DiaRD-Hpykl	20,8	N/A	N/A
**141**	Adenoid Tissue	DNA	100	DiaRex Tissue Kit	TS-6826	DiaRD-Hpykl	19,9	N/A	N/A
**142**	Adenoid Tissue	DNA	100	DiaRex Tissue Kit	TS-6826	DiaRD-Hpykl	20,3	N/A	N/A
**143**	Adenoid Tissue	DNA	100	DiaRex Tissue Kit	TS-6826	DiaRD-Hpykl	19,5	N/A	N/A
**144**	Adenoid Tissue	DNA	100	DiaRex Tissue Kit	TS-6826	DiaRD-Hpykl	20,2	N/A	N/A
**145**	Adenoid Tissue	DNA	100	DiaRex Tissue Kit	TS-6826	DiaRD-Hpykl	19,1	N/A	N/A
**146**	Adenoid Tissue	DNA	100	DiaRex Tissue Kit	TS-6826	DiaRD-Hpykl	19,0	N/A	N/A
**147**	Adenoid Tissue	DNA	100	DiaRex Tissue Kit	TS-6826	DiaRD-Hpykl	21,4	N/A	N/A
**148**	Adenoid Tissue	DNA	100	DiaRex Tissue Kit	TS-6826	DiaRD-Hpykl	21,6	N/A	N/A
**149**	Adenoid Tissue	DNA	100	DiaRex Tissue Kit	TS-6826	DiaRD-Hpykl	20,7	N/A	N/A
**150**	Adenoid Tissue	DNA	100	DiaRex Tissue Kit	TS-6826	DiaRD-Hpykl	19,2	N/A	N/A
**151**	Adenoid Tissue	DNA	100	DiaRex Tissue Kit	TS-6826	DiaRD-Hpykl	21,3	N/A	N/A
**152**	Adenoid Tissue	DNA	100	DiaRex Tissue Kit	TS-6826	DiaRD-Hpykl	19,8	N/A	N/A
**153**	Adenoid Tissue	DNA	100	DiaRex Tissue Kit	TS-6826	DiaRD-Hpykl	20,8	N/A	N/A
**154**	Adenoid Tissue	DNA	100	DiaRex Tissue Kit	TS-6826	DiaRD-Hpykl	21,7	N/A	N/A
**155**	Adenoid Tissue	DNA	100	DiaRex Tissue Kit	TS-6826	DiaRD-Hpykl	20,9	N/A	N/A
**156**	Adenoid Tissue	DNA	100	DiaRex Tissue Kit	TS-6826	DiaRD-Hpykl	19,9	N/A	N/A
**157**	Adenoid Tissue	DNA	100	DiaRex Tissue Kit	TS-6826	DiaRD-Hpykl	20,6	N/A	N/A
**158**	Adenoid Tissue	DNA	100	DiaRex Tissue Kit	TS-6826	DiaRD-Hpykl	19,8	N/A	N/A
**159**	Adenoid Tissue	DNA	100	DiaRex Tissue Kit	TS-6826	DiaRD-Hpykl	23,8	N/A	N/A
**160**	Adenoid Tissue	DNA	100	DiaRex Tissue Kit	TS-6826	DiaRD-Hpykl	20,0	N/A	N/A
**161**	Adenoid Tissue	DNA	100	DiaRex Tissue Kit	TS-6826	DiaRD-Hpykl	20,4	N/A	N/A
**162**	Adenoid Tissue	DNA	100	DiaRex Tissue Kit	TS-6826	DiaRD-Hpykl	20,5	N/A	N/A
**163**	Adenoid Tissue	DNA	100	DiaRex Tissue Kit	TS-6826	DiaRD-Hpykl	20,5	N/A	N/A
**164**	Adenoid Tissue	DNA	100	DiaRex Tissue Kit	TS-6826	DiaRD-Hpykl	20,5	N/A	N/A
**165**	Adenoid Tissue	DNA	100	DiaRex Tissue Kit	TS-6826	DiaRD-Hpykl	23,3	N/A	N/A
**166**	Adenoid Tissue	DNA	100	DiaRex Tissue Kit	TS-6826	DiaRD-Hpykl	22,2	N/A	N/A
**167**	Adenoid Tissue	DNA	100	DiaRex Tissue Kit	TS-6826	DiaRD-Hpykl	22,0	N/A	N/A

**168**	Adenoid Tissue	DNA	100	DiaRex Tissue Kit	TS-6826	DiaRD-Hpykl	20,1	N/A	N/A
**169**	Adenoid Tissue	DNA	100	DiaRex Tissue Kit	TS-6826	DiaRD-Hpykl	21,0	N/A	N/A
**170**	Adenoid Tissue	DNA	100	DiaRex Tissue Kit	TS-6826	DiaRD-Hpykl	21,7	N/A	N/A
**171**	Adenoid Tissue	DNA	100	DiaRex Tissue Kit	TS-6826	DiaRD-Hpykl	20,7	N/A	N/A
**172**	Adenoid Tissue	DNA	100	DiaRex Tissue Kit	TS-6826	DiaRD-Hpykl	21,2	N/A	N/A
**173**	Adenoid Tissue	DNA	100	DiaRex Tissue Kit	TS-6826	DiaRD-Hpykl	21,0	N/A	N/A
**174**	Adenoid Tissue	DNA	100	DiaRex Tissue Kit	TS-6826	DiaRD-Hpykl	19,5	N/A	N/A
**175**	Adenoid Tissue	DNA	100	DiaRex Tissue Kit	TS-6826	DiaRD-Hpykl	20,3	N/A	N/A
**176**	Adenoid Tissue	DNA	100	DiaRex Tissue Kit	TS-6826	DiaRD-Hpykl	19,6	N/A	N/A
**177**	Adenoid Tissue	DNA	100	DiaRex Tissue Kit	TS-6826	DiaRD-Hpykl	23,9	N/A	N/A
**178**	Adenoid Tissue	DNA	100	DiaRex Tissue Kit	TS-6826	DiaRD-Hpykl	20,4	N/A	N/A
**179**	Adenoid Tissue	DNA	100	DiaRex Tissue Kit	TS-6826	DiaRD-Hpykl	21,7	N/A	N/A
**180**	Adenoid Tissue	DNA	100	DiaRex Tissue Kit	TS-6826	DiaRD-Hpykl	21,8	N/A	N/A
**181**	Adenoid Tissue	DNA	100	DiaRex Tissue Kit	TS-6826	DiaRD-Hpykl	20,2	N/A	N/A
**182**	Adenoid Tissue	DNA	100	DiaRex Tissue Kit	TS-6826	DiaRD-Hpykl	21,9	N/A	N/A
**183**	Adenoid Tissue	DNA	100	DiaRex Tissue Kit	TS-6826	DiaRD-Hpykl	20,0	N/A	N/A
**184**	Adenoid Tissue	DNA	100	DiaRex Tissue Kit	TS-6826	DiaRD-Hpykl	20,8	N/A	N/A
**185**	Adenoid Tissue	DNA	100	DiaRex Tissue Kit	TS-6826	DiaRD-Hpykl	21,5	N/A	N/A
**186**	Adenoid Tissue	DNA	100	DiaRex Tissue Kit	TS-6826	DiaRD-Hpykl	19,4	N/A	N/A
**187**	Adenoid Tissue	DNA	100	DiaRex Tissue Kit	TS-6826	DiaRD-Hpykl	21,6	N/A	N/A
**188**	Adenoid Tissue	DNA	100	DiaRex Tissue Kit	TS-6826	DiaRD-Hpykl	22,5	N/A	N/A
**189**	Adenoid Tissue	DNA	100	DiaRex Tissue Kit	TS-6826	DiaRD-Hpykl	21,0	N/A	N/A
**190**	Adenoid Tissue	DNA	100	DiaRex Tissue Kit	TS-6826	DiaRD-Hpykl	20,5	N/A	N/A
**191**	Adenoid Tissue	DNA	100	DiaRex Tissue Kit	TS-6826	DiaRD-Hpykl	20,2	N/A	N/A
**192**	Adenoid Tissue	DNA	100	DiaRex Tissue Kit	TS-6826	DiaRD-Hpykl	21,2	N/A	N/A
**193**	Adenoid Tissue	DNA	100	DiaRex Tissue Kit	TS-6826	DiaRD-Hpykl	20,6	N/A	N/A
**194**	Adenoid Tissue	DNA	100	DiaRex Tissue Kit	TS-6826	DiaRD-Hpykl	26,0	N/A	N/A
**195**	Adenoid Tissue	DNA	100	DiaRex Tissue Kit	TS-6826	DiaRD-Hpykl	21,3	N/A	N/A
**196**	Adenoid Tissue	DNA	100	DiaRex Tissue Kit	TS-6826	DiaRD-Hpykl	20,7	N/A	N/A
**197**	Adenoid Tissue	DNA	100	DiaRex Tissue Kit	TS-6826	DiaRD-Hpykl	22,2	N/A	N/A
**198**	Adenoid Tissue	DNA	100	DiaRex Tissue Kit	TS-6826	DiaRD-Hpykl	20,3	N/A	N/A
**199**	Adenoid Tissue	DNA	100	DiaRex Tissue Kit	TS-6826	DiaRD-Hpykl	19,5	N/A	N/A
**200**	Adenoid Tissue	DNA	100	DiaRex Tissue Kit	TS-6826	DiaRD-Hpykl	20,3	N/A	N/A
**201**	Middle Ear Fluid	DNA	100	DiaRex Blood Kit	BLD-5295	DiaRD-Hpykl	26,8	N/A	N/A

**202**	Middle Ear Fluid	DNA	100	DiaRex Blood Kit	BLD-5295	DiaRD-Hpykl	26,5	N/A	N/A
**203**	Middle Ear Fluid	DNA	100	DiaRex Blood Kit	BLD-5295	DiaRD-Hpykl	21,1	N/A	N/A
**204**	Middle Ear Fluid	DNA	100	DiaRex Blood Kit	BLD-5295	DiaRD-Hpykl	26,5	N/A	N/A
**205**	Middle Ear Fluid	DNA	100	DiaRex Blood Kit	BLD-5295	DiaRD-Hpykl	24,9	N/A	N/A
**206**	Middle Ear Fluid	DNA	100	DiaRex Blood Kit	BLD-5295	DiaRD-Hpykl	27,4	N/A	N/A
**207**	Middle Ear Fluid	DNA	100	DiaRex Blood Kit	BLD-5295	DiaRD-Hpykl	27,2	N/A	N/A
**208**	Middle Ear Fluid	DNA	100	DiaRex Blood Kit	BLD-5295	DiaRD-Hpykl	28,4	N/A	N/A
**209**	Middle Ear Fluid	DNA	100	DiaRex Blood Kit	BLD-5295	DiaRD-Hpykl	27,4	N/A	N/A
**210**	Middle Ear Fluid	DNA	100	DiaRex Blood Kit	BLD-5295	DiaRD-Hpykl	26,1	N/A	N/A
**211**	Middle Ear Fluid	DNA	100	DiaRex Blood Kit	BLD-5295	DiaRD-Hpykl	30,1	N/A	N/A
**212**	Middle Ear Fluid	DNA	100	DiaRex Blood Kit	BLD-5295	DiaRD-Hpykl	28,3	N/A	N/A
**213**	Middle Ear Fluid	DNA	100	DiaRex Blood Kit	BLD-5295	DiaRD-Hpykl	29,4	N/A	N/A
**214**	Middle Ear Fluid	DNA	100	DiaRex Blood Kit	BLD-5295	DiaRD-Hpykl	27,7	N/A	N/A
**215**	Middle Ear Fluid	DNA	100	DiaRex Blood Kit	BLD-5295	DiaRD-Hpykl	27,4	N/A	N/A
**216**	Middle Ear Fluid	DNA	100	DiaRex Blood Kit	BLD-5295	DiaRD-Hpykl	28,5	N/A	N/A
**217**	Middle Ear Fluid	DNA	100	DiaRex Blood Kit	BLD-5295	DiaRD-Hpykl	28,1	N/A	N/A
**218**	Middle Ear Fluid	DNA	100	DiaRex Blood Kit	BLD-5295	DiaRD-Hpykl	27,8	N/A	N/A
**219**	Middle Ear Fluid	DNA	100	DiaRex Blood Kit	BLD-5295	DiaRD-Hpykl	29,1	N/A	N/A
**220**	Middle Ear Fluid	DNA	100	DiaRex Blood Kit	BLD-5295	DiaRD-Hpykl	28,9	N/A	N/A
**221**	Middle Ear Fluid	DNA	100	DiaRex Blood Kit	BLD-5295	DiaRD-Hpykl	23,8	N/A	N/A
**222**	Middle Ear Fluid	DNA	100	DiaRex Blood Kit	BLD-5295	DiaRD-Hpykl	28,2	N/A	N/A
**223**	Middle Ear Fluid	DNA	100	DiaRex Blood Kit	BLD-5295	DiaRD-Hpykl	26,4	N/A	N/A
**224**	Middle Ear Fluid	DNA	100	DiaRex Blood Kit	BLD-5295	DiaRD-Hpykl	26,4	N/A	N/A
**225**	Middle Ear Fluid	DNA	100	DiaRex Blood Kit	BLD-5295	DiaRD-Hpykl	26,5	N/A	N/A
**226**	Middle Ear Fluid	DNA	100	DiaRex Blood Kit	BLD-5295	DiaRD-Hpykl	29,0	N/A	N/A
**227**	Middle Ear Fluid	DNA	100	DiaRex Blood Kit	BLD-5295	DiaRD-Hpykl	29,0	N/A	N/A
**228**	Middle Ear Fluid	DNA	100	DiaRex Blood Kit	BLD-5295	DiaRD-Hpykl	31,3	N/A	N/A
**229**	Middle Ear Fluid	DNA	100	DiaRex Blood Kit	BLD-5295	DiaRD-Hpykl	26,8	N/A	N/A
**230**	Middle Ear Fluid	DNA	100	DiaRex Blood Kit	BLD-5295	DiaRD-Hpykl	29,1	N/A	N/A
**231**	Middle Ear Fluid	DNA	100	DiaRex Blood Kit	BLD-5295	DiaRD-Hpykl	24,8	N/A	N/A
**232**	Middle Ear Fluid	DNA	100	DiaRex Blood Kit	BLD-5295	DiaRD-Hpykl	25,6	N/A	N/A
**233**	Middle Ear Fluid	DNA	100	DiaRex Blood Kit	BLD-5295	DiaRD-Hpykl	30,0	N/A	N/A
**234**	Middle Ear Fluid	DNA	100	DiaRex Blood Kit	BLD-5295	DiaRD-Hpykl	27,1	N/A	N/A
**235**	Middle Ear Fluid	DNA	100	DiaRex Blood Kit	BLD-5295	DiaRD-Hpykl	25,4	N/A	N/A

**236**	Middle Ear Fluid	DNA	100	DiaRex Blood Kit	BLD-5295	DiaRD-Hpykl	34,2	N/A	N/A
**237**	Middle Ear Fluid	DNA	100	DiaRex Blood Kit	BLD-5295	DiaRD-Hpykl	25,6	N/A	N/A
**238**	Middle Ear Fluid	DNA	100	DiaRex Blood Kit	BLD-5295	DiaRD-Hpykl	29,3	N/A	N/A
**239**	Middle Ear Fluid	DNA	100	DiaRex Blood Kit	BLD-5295	DiaRD-Hpykl	26,8	N/A	N/A
**240**	Middle Ear Fluid	DNA	100	DiaRex Blood Kit	BLD-5295	DiaRD-Hpykl	26,3	N/A	N/A
**241**	Middle Ear Fluid	DNA	100	DiaRex Blood Kit	BLD-5295	DiaRD-Hpykl	23,7	N/A	N/A
**242**	Middle Ear Fluid	DNA	100	DiaRex Blood Kit	BLD-5295	DiaRD-Hpykl	26,8	N/A	N/A
**243**	Middle Ear Fluid	DNA	100	DiaRex Blood Kit	BLD-5295	DiaRD-Hpykl	29,4	N/A	N/A
**244**	Middle Ear Fluid	DNA	100	DiaRex Blood Kit	BLD-5295	DiaRD-Hpykl	27,0	N/A	N/A
**245**	Middle Ear Fluid	DNA	100	DiaRex Blood Kit	BLD-5295	DiaRD-Hpykl	24,5	N/A	N/A
**246**	Middle Ear Fluid	DNA	100	DiaRex Blood Kit	BLD-5295	DiaRD-Hpykl	24,0	N/A	N/A
**247**	Middle Ear Fluid	DNA	100	DiaRex Blood Kit	BLD-5295	DiaRD-Hpykl	28,5	N/A	N/A
**248**	Middle Ear Fluid	DNA	100	DiaRex Blood Kit	BLD-5295	DiaRD-Hpykl	27,3	N/A	N/A
**249**	Middle Ear Fluid	DNA	100	DiaRex Blood Kit	BLD-5295	DiaRD-Hpykl	27,6	N/A	N/A
**250**	Middle Ear Fluid	DNA	100	DiaRex Blood Kit	BLD-5295	DiaRD-Hpykl	28,9	N/A	N/A
**251**	Middle Ear Fluid	DNA	100	DiaRex Blood Kit	BLD-5295	DiaRD-Hpykl	24,6	N/A	N/A
**252**	Middle Ear Fluid	DNA	100	DiaRex Blood Kit	BLD-5295	DiaRD-Hpykl	21,8	N/A	N/A
**253**	Middle Ear Fluid	DNA	100	DiaRex Blood Kit	BLD-5295	DiaRD-Hpykl	22,0	N/A	N/A
**254**	Middle Ear Fluid	DNA	100	DiaRex Blood Kit	BLD-5295	DiaRD-Hpykl	22,7	N/A	N/A
**255**	Middle Ear Fluid	DNA	100	DiaRex Blood Kit	BLD-5295	DiaRD-Hpykl	21,2	N/A	N/A
**256**	Middle Ear Fluid	DNA	100	DiaRex Blood Kit	BLD-5295	DiaRD-Hpykl	26,1	N/A	N/A
**257**	Middle Ear Fluid	DNA	100	DiaRex Blood Kit	BLD-5295	DiaRD-Hpykl	30,1	N/A	N/A
**258**	Middle Ear Fluid	DNA	100	DiaRex Blood Kit	BLD-5295	DiaRD-Hpykl	25,8	N/A	N/A
**259**	Middle Ear Fluid	DNA	100	DiaRex Blood Kit	BLD-5295	DiaRD-Hpykl	22,2	N/A	N/A
**260**	Middle Ear Fluid	DNA	100	DiaRex Blood Kit	BLD-5295	DiaRD-Hpykl	21,6	N/A	N/A
**261**	Middle Ear Fluid	DNA	100	DiaRex Blood Kit	BLD-5295	DiaRD-Hpykl	24,0	N/A	N/A
**262**	Middle Ear Fluid	DNA	100	DiaRex Blood Kit	BLD-5295	DiaRD-Hpykl	23,1	N/A	N/A
**263**	Middle Ear Fluid	DNA	100	DiaRex Blood Kit	BLD-5295	DiaRD-Hpykl	22,2	N/A	N/A
**264**	Middle Ear Fluid	DNA	100	DiaRex Blood Kit	BLD-5295	DiaRD-Hpykl	27,3	N/A	N/A
**265**	Middle Ear Fluid	DNA	100	DiaRex Blood Kit	BLD-5295	DiaRD-Hpykl	27,4	N/A	N/A
**266**	Middle Ear Fluid	DNA	100	DiaRex Blood Kit	BLD-5295	DiaRD-Hpykl	30,6	N/A	N/A
**267**	Middle Ear Fluid	DNA	100	DiaRex Blood Kit	BLD-5295	DiaRD-Hpykl	27,0	N/A	N/A
**268**	Middle Ear Fluid	DNA	100	DiaRex Blood Kit	BLD-5295	DiaRD-Hpykl	24,3	N/A	N/A
**269**	Middle Ear Fluid	DNA	100	DiaRex Blood Kit	BLD-5295	DiaRD-Hpykl	28,0	N/A	N/A

**270**	Middle Ear Fluid	DNA	100	DiaRex Blood Kit	BLD-5295	DiaRD-Hpykl	22,1	N/A	N/A
**271**	Middle Ear Fluid	DNA	100	DiaRex Blood Kit	BLD-5295	DiaRD-Hpykl	28,0	N/A	N/A
**272**	Middle Ear Fluid	DNA	100	DiaRex Blood Kit	BLD-5295	DiaRD-Hpykl	29,4	N/A	N/A
**273**	Middle Ear Fluid	DNA	100	DiaRex Blood Kit	BLD-5295	DiaRD-Hpykl	28,1	N/A	N/A
**274**	Middle Ear Fluid	DNA	100	DiaRex Blood Kit	BLD-5295	DiaRD-Hpykl	28,6	N/A	N/A
**275**	Middle Ear Fluid	DNA	100	DiaRex Blood Kit	BLD-5295	DiaRD-Hpykl	21,7	N/A	N/A
**276**	Middle Ear Fluid	DNA	100	DiaRex Blood Kit	BLD-5295	DiaRD-Hpykl	29,0	N/A	N/A
**277**	Middle Ear Fluid	DNA	100	DiaRex Blood Kit	BLD-5295	DiaRD-Hpykl	23,6	N/A	N/A
**278**	Middle Ear Fluid	DNA	100	DiaRex Blood Kit	BLD-5295	DiaRD-Hpykl	22,5	N/A	N/A
**279**	Middle Ear Fluid	DNA	100	DiaRex Blood Kit	BLD-5295	DiaRD-Hpykl	23,8	N/A	N/A
**280**	Middle Ear Fluid	DNA	100	DiaRex Blood Kit	BLD-5295	DiaRD-Hpykl	26,1	N/A	N/A
**281**	Middle Ear Fluid	DNA	100	DiaRex Blood Kit	BLD-5295	DiaRD-Hpykl	29,7	N/A	N/A
**282**	Middle Ear Fluid	DNA	100	DiaRex Blood Kit	BLD-5295	DiaRD-Hpykl	28,8	N/A	N/A
**283**	Middle Ear Fluid	DNA	100	DiaRex Blood Kit	BLD-5295	DiaRD-Hpykl	27,5	N/A	N/A
**284**	Middle Ear Fluid	DNA	100	DiaRex Blood Kit	BLD-5295	DiaRD-Hpykl	24,8	N/A	N/A
**285**	Middle Ear Fluid	DNA	100	DiaRex Blood Kit	BLD-5295	DiaRD-Hpykl	23,1	N/A	N/A
**286**	Middle Ear Fluid	DNA	100	DiaRex Blood Kit	BLD-5295	DiaRD-Hpykl	21,0	N/A	N/A
**287**	Middle Ear Fluid	DNA	100	DiaRex Blood Kit	BLD-5295	DiaRD-Hpykl	26,3	N/A	N/A
**288**	Middle Ear Fluid	DNA	100	DiaRex Blood Kit	BLD-5295	DiaRD-Hpykl	24,4	N/A	N/A
**289**	Middle Ear Fluid	DNA	100	DiaRex Blood Kit	BLD-5295	DiaRD-Hpykl	24,9	N/A	N/A
**290**	Middle Ear Fluid	DNA	100	DiaRex Blood Kit	BLD-5295	DiaRD-Hpykl	30,1	N/A	N/A
**291**	Middle Ear Fluid	DNA	100	DiaRex Blood Kit	BLD-5295	DiaRD-Hpykl	24,5	N/A	N/A
**292**	Middle Ear Fluid	DNA	100	DiaRex Blood Kit	BLD-5295	DiaRD-Hpykl	30,0	N/A	N/A
**293**	Middle Ear Fluid	DNA	100	DiaRex Blood Kit	BLD-5295	DiaRD-Hpykl	28,7	N/A	N/A
**294**	Middle Ear Fluid	DNA	100	DiaRex Blood Kit	BLD-5295	DiaRD-Hpykl	24,5	N/A	N/A
**295**	Middle Ear Fluid	DNA	100	DiaRex Blood Kit	BLD-5295	DiaRD-Hpykl	24,5	N/A	N/A
**296**	Middle Ear Fluid	DNA	100	DiaRex Blood Kit	BLD-5295	DiaRD-Hpykl	27,9	N/A	N/A
**297**	Middle Ear Fluid	DNA	100	DiaRex Blood Kit	BLD-5295	DiaRD-Hpykl	26,6	N/A	N/A
**298**	Middle Ear Fluid	DNA	100	DiaRex Blood Kit	BLD-5295	DiaRD-Hpykl	28,9	N/A	N/A
**299**	Middle Ear Fluid	DNA	100	DiaRex Blood Kit	BLD-5295	DiaRD-Hpykl	24,3	N/A	N/A
**300**	Middle Ear Fluid	DNA	100	DiaRex Blood Kit	BLD-5295	DiaRD-Hpykl	30,5	N/A	N/A
**301**	Middle Ear Fluid	DNA	100	DiaRex Blood Kit	BLD-5295	DiaRD-Hpykl	24,9	N/A	N/A
**302**	Middle Ear Fluid	DNA	100	DiaRex Blood Kit	BLD-5295	DiaRD-Hpykl	25,7	N/A	N/A
**303**	Middle Ear Fluid	DNA	100	DiaRex Blood Kit	BLD-5295	DiaRD-Hpykl	29,8	N/A	N/A

**304**	Middle Ear Fluid	DNA	100	DiaRex Blood Kit	BLD-5295	DiaRD-Hpykl	27,1	N/A	N/A
**305**	Middle Ear Fluid	DNA	100	DiaRex Blood Kit	BLD-5295	DiaRD-Hpykl	30,3	N/A	N/A
**306**	Middle Ear Fluid	DNA	100	DiaRex Blood Kit	BLD-5295	DiaRD-Hpykl	28,1	N/A	N/A
**307**	Middle Ear Fluid	DNA	100	DiaRex Blood Kit	BLD-5295	DiaRD-Hpykl	29,8	N/A	N/A
**308**	Middle Ear Fluid	DNA	100	DiaRex Blood Kit	BLD-5295	DiaRD-Hpykl	28,7	N/A	N/A
**309**	Middle Ear Fluid	DNA	100	DiaRex Blood Kit	BLD-5295	DiaRD-Hpykl	27,9	N/A	N/A
**310**	Middle Ear Fluid	DNA	100	DiaRex Blood Kit	BLD-5295	DiaRD-Hpykl	28,2	N/A	N/A
**311**	Middle Ear Fluid	DNA	100	DiaRex Blood Kit	BLD-5295	DiaRD-Hpykl	28,8	N/A	N/A
**312**	Middle Ear Fluid	DNA	100	DiaRex Blood Kit	BLD-5295	DiaRD-Hpykl	25,1	N/A	N/A
**313**	Middle Ear Fluid	DNA	100	DiaRex Blood Kit	BLD-5295	DiaRD-Hpykl	25,1	N/A	N/A
**314**	Middle Ear Fluid	DNA	100	DiaRex Blood Kit	BLD-5295	DiaRD-Hpykl	27,5	N/A	N/A
**315**	Middle Ear Fluid	DNA	100	DiaRex Blood Kit	BLD-5295	DiaRD-Hpykl	29,3	N/A	N/A
**316**	Middle Ear Fluid	DNA	100	DiaRex Blood Kit	BLD-5295	DiaRD-Hpykl	24,3	N/A	N/A
**317**	Middle Ear Fluid	DNA	100	DiaRex Blood Kit	BLD-5295	DiaRD-Hpykl	22,7	N/A	N/A
**318**	Middle Ear Fluid	DNA	100	DiaRex Blood Kit	BLD-5295	DiaRD-Hpykl	26,3	N/A	N/A
**319**	Middle Ear Fluid	DNA	100	DiaRex Blood Kit	BLD-5295	DiaRD-Hpykl	24,0	N/A	N/A
**320**	Middle Ear Fluid	DNA	100	DiaRex Blood Kit	BLD-5295	DiaRD-Hpykl	25,1	N/A	N/A
**321**	Middle Ear Fluid	DNA	100	DiaRex Blood Kit	BLD-5295	DiaRD-Hpykl	28,0	N/A	N/A
**322**	Middle Ear Fluid	DNA	100	DiaRex Blood Kit	BLD-5295	DiaRD-Hpykl	30,4	N/A	N/A
**323**	Middle Ear Fluid	DNA	100	DiaRex Blood Kit	BLD-5295	DiaRD-Hpykl	28,2	N/A	N/A
**324**	Middle Ear Fluid	DNA	100	DiaRex Blood Kit	BLD-5295	DiaRD-Hpykl	25,0	N/A	N/A
**325**	Middle Ear Fluid	DNA	100	DiaRex Blood Kit	BLD-5295	DiaRD-Hpykl	22,4	N/A	N/A
**326**	Middle Ear Fluid	DNA	100	DiaRex Blood Kit	BLD-5295	DiaRD-Hpykl	25,3	N/A	N/A
**327**	Middle Ear Fluid	DNA	100	DiaRex Blood Kit	BLD-5295	DiaRD-Hpykl	25,9	N/A	N/A
**328**	Middle Ear Fluid	DNA	100	DiaRex Blood Kit	BLD-5295	DiaRD-Hpykl	28,2	N/A	N/A
**329**	Middle Ear Fluid	DNA	100	DiaRex Blood Kit	BLD-5295	DiaRD-Hpykl	29,1	N/A	N/A
**330**	Middle Ear Fluid	DNA	100	DiaRex Blood Kit	BLD-5295	DiaRD-Hpykl	22,2	N/A	N/A
**331**	Middle Ear Fluid	DNA	100	DiaRex Blood Kit	BLD-5295	DiaRD-Hpykl	31,2	N/A	N/A
**332**	Middle Ear Fluid	DNA	100	DiaRex Blood Kit	BLD-5295	DiaRD-Hpykl	30,6	N/A	N/A
**333**	Middle Ear Fluid	DNA	100	DiaRex Blood Kit	BLD-5295	DiaRD-Hpykl	28,7	N/A	N/A
**334**	Middle Ear Fluid	DNA	100	DiaRex Blood Kit	BLD-5295	DiaRD-Hpykl	26,3	N/A	N/A
**335**	Middle Ear Fluid	DNA	100	DiaRex Blood Kit	BLD-5295	DiaRD-Hpykl	29,7	N/A	N/A
**336**	Middle Ear Fluid	DNA	100	DiaRex Blood Kit	BLD-5295	DiaRD-Hpykl	23,0	N/A	N/A
**337**	Middle Ear Fluid	DNA	100	DiaRex Blood Kit	BLD-5295	DiaRD-Hpykl	28,2	N/A	N/A

**338**	Middle Ear Fluid	DNA	100	DiaRex Blood Kit	BLD-5295	DiaRD-Hpykl	25,5	N/A	N/A
**339**	Middle Ear Fluid	DNA	100	DiaRex Blood Kit	BLD-5295	DiaRD-Hpykl	22,0	N/A	N/A
**340**	Middle Ear Fluid	DNA	100	DiaRex Blood Kit	BLD-5295	DiaRD-Hpykl	29,4	N/A	N/A
**341**	Middle Ear Fluid	DNA	100	DiaRex Blood Kit	BLD-5295	DiaRD-Hpykl	23,6	N/A	N/A
**342**	Middle Ear Fluid	DNA	100	DiaRex Blood Kit	BLD-5295	DiaRD-Hpykl	28,5	N/A	N/A
**343**	Middle Ear Fluid	DNA	100	DiaRex Blood Kit	BLD-5295	DiaRD-Hpykl	25,1	N/A	N/A
**344**	Middle Ear Fluid	DNA	100	DiaRex Blood Kit	BLD-5295	DiaRD-Hpykl	28,3	N/A	N/A
**345**	Middle Ear Fluid	DNA	100	DiaRex Blood Kit	BLD-5295	DiaRD-Hpykl	30,2	N/A	N/A
**346**	Middle Ear Fluid	DNA	100	DiaRex Blood Kit	BLD-5295	DiaRD-Hpykl	29,3	N/A	N/A
**347**	Middle Ear Fluid	DNA	100	DiaRex Blood Kit	BLD-5295	DiaRD-Hpykl	26,7	N/A	N/A
**348**	Middle Ear Fluid	DNA	100	DiaRex Blood Kit	BLD-5295	DiaRD-Hpykl	23,5	N/A	N/A
**349**	Middle Ear Fluid	DNA	100	DiaRex Blood Kit	BLD-5295	DiaRD-Hpykl	25,5	N/A	N/A
**350**	Middle Ear Fluid	DNA	100	DiaRex Blood Kit	BLD-5295	DiaRD-Hpykl	28,1	N/A	N/A
**351**	Middle Ear Fluid	DNA	100	DiaRex Blood Kit	BLD-5295	DiaRD-Hpykl	29,8	N/A	N/A
**352**	Middle Ear Fluid	DNA	100	DiaRex Blood Kit	BLD-5295	DiaRD-Hpykl	24,7	N/A	N/A
**353**	Middle Ear Fluid	DNA	100	DiaRex Blood Kit	BLD-5295	DiaRD-Hpykl	28,9	N/A	N/A
**354**	Middle Ear Fluid	DNA	100	DiaRex Blood Kit	BLD-5295	DiaRD-Hpykl	28,9	N/A	N/A
**355**	Middle Ear Fluid	DNA	100	DiaRex Blood Kit	BLD-5295	DiaRD-Hpykl	28,8	N/A	N/A
**356**	Middle Ear Fluid	DNA	100	DiaRex Blood Kit	BLD-5295	DiaRD-Hpykl	27,2	N/A	N/A
**357**	Middle Ear Fluid	DNA	100	DiaRex Blood Kit	BLD-5295	DiaRD-Hpykl	30,0	N/A	N/A
**358**	Middle Ear Fluid	DNA	100	DiaRex Blood Kit	BLD-5295	DiaRD-Hpykl	22,1	N/A	N/A
**359**	Middle Ear Fluid	DNA	100	DiaRex Blood Kit	BLD-5295	DiaRD-Hpykl	23,1	N/A	N/A
**360**	Middle Ear Fluid	DNA	100	DiaRex Blood Kit	BLD-5295	DiaRD-Hpykl	28,8	N/A	N/A
**361**	Middle Ear Fluid	DNA	100	DiaRex Blood Kit	BLD-5295	DiaRD-Hpykl	28,3	N/A	N/A
**362**	Middle Ear Fluid	DNA	100	DiaRex Blood Kit	BLD-5295	DiaRD-Hpykl	31,2	N/A	N/A
**363**	Middle Ear Fluid	DNA	100	DiaRex Blood Kit	BLD-5295	DiaRD-Hpykl	29,5	N/A	N/A
**364**	Middle Ear Fluid	DNA	100	DiaRex Blood Kit	BLD-5295	DiaRD-Hpykl	28,3	N/A	N/A
**365**	Middle Ear Fluid	DNA	100	DiaRex Blood Kit	BLD-5295	DiaRD-Hpykl	22,5	N/A	N/A
**366**	Middle Ear Fluid	DNA	100	DiaRex Blood Kit	BLD-5295	DiaRD-Hpykl	25,6	N/A	N/A
**367**	Middle Ear Fluid	DNA	100	DiaRex Blood Kit	BLD-5295	DiaRD-Hpykl	22,0	N/A	N/A
**368**	Middle Ear Fluid	DNA	100	DiaRex Blood Kit	BLD-5295	DiaRD-Hpykl	23,6	N/A	N/A
**369**	Middle Ear Fluid	DNA	100	DiaRex Blood Kit	BLD-5295	DiaRD-Hpykl	27,0	N/A	N/A
**370**	Middle Ear Fluid	DNA	100	DiaRex Blood Kit	BLD-5295	DiaRD-Hpykl	25,4	N/A	N/A
**371**	Middle Ear Fluid	DNA	100	DiaRex Blood Kit	BLD-5295	DiaRD-Hpykl	24,1	N/A	N/A

**372**	Middle Ear Fluid	DNA	100	DiaRex Blood Kit	BLD-5295	DiaRD-Hpykl	28,5	N/A	N/A
**373**	Middle Ear Fluid	DNA	100	DiaRex Blood Kit	BLD-5295	DiaRD-Hpykl	29,4	N/A	N/A
**374**	Middle Ear Fluid	DNA	100	DiaRex Blood Kit	BLD-5295	DiaRD-Hpykl	27,8	N/A	N/A
**375**	Middle Ear Fluid	DNA	100	DiaRex Blood Kit	BLD-5295	DiaRD-Hpykl	29,4	N/A	N/A
**376**	Middle Ear Fluid	DNA	100	DiaRex Blood Kit	BLD-5295	DiaRD-Hpykl	24,5	N/A	N/A
**377**	Middle Ear Fluid	DNA	100	DiaRex Blood Kit	BLD-5295	DiaRD-Hpykl	24,2	N/A	N/A
**378**	Middle Ear Fluid	DNA	100	DiaRex Blood Kit	BLD-5295	DiaRD-Hpykl	29,5	N/A	N/A
**379**	Middle Ear Fluid	DNA	100	DiaRex Blood Kit	BLD-5295	DiaRD-Hpykl	25,0	N/A	N/A
**380**	Middle Ear Fluid	DNA	100	DiaRex Blood Kit	BLD-5295	DiaRD-Hpykl	25,0	N/A	N/A
**381**	Middle Ear Fluid	DNA	100	DiaRex Blood Kit	BLD-5295	DiaRD-Hpykl	24,3	N/A	N/A
**382**	Middle Ear Fluid	DNA	100	DiaRex Blood Kit	BLD-5295	DiaRD-Hpykl	28,3	N/A	N/A
**383**	Middle Ear Fluid	DNA	100	DiaRex Blood Kit	BLD-5295	DiaRD-Hpykl	26,4	N/A	N/A
**384**	Middle Ear Fluid	DNA	100	DiaRex Blood Kit	BLD-5295	DiaRD-Hpykl	27,6	N/A	N/A
**385**	Middle Ear Fluid	DNA	100	DiaRex Blood Kit	BLD-5295	DiaRD-Hpykl	25,3	N/A	N/A
**386**	Middle Ear Fluid	DNA	100	DiaRex Blood Kit	BLD-5295	DiaRD-Hpykl	25,5	N/A	N/A
**387**	Middle Ear Fluid	DNA	100	DiaRex Blood Kit	BLD-5295	DiaRD-Hpykl	28,7	N/A	N/A
**388**	Middle Ear Fluid	DNA	100	DiaRex Blood Kit	BLD-5295	DiaRD-Hpykl	26,5	N/A	N/A
**389**	Middle Ear Fluid	DNA	100	DiaRex Blood Kit	BLD-5295	DiaRD-Hpykl	26,3	N/A	N/A
**390**	Middle Ear Fluid	DNA	100	DiaRex Blood Kit	BLD-5295	DiaRD-Hpykl	27,8	N/A	N/A
**391**	Middle Ear Fluid	DNA	100	DiaRex Blood Kit	BLD-5295	DiaRD-Hpykl	23,3	N/A	N/A
**392**	Middle Ear Fluid	DNA	100	DiaRex Blood Kit	BLD-5295	DiaRD-Hpykl	30,1	N/A	N/A
**393**	Middle Ear Fluid	DNA	100	DiaRex Blood Kit	BLD-5295	DiaRD-Hpykl	29,1	N/A	N/A
**394**	Middle Ear Fluid	DNA	100	DiaRex Blood Kit	BLD-5295	DiaRD-Hpykl	29,1	N/A	N/A
**395**	Middle Ear Fluid	DNA	100	DiaRex Blood Kit	BLD-5295	DiaRD-Hpykl	28,4	N/A	N/A
**396**	Middle Ear Fluid	DNA	100	DiaRex Blood Kit	BLD-5295	DiaRD-Hpykl	28,7	N/A	N/A
**397**	Middle Ear Fluid	DNA	100	DiaRex Blood Kit	BLD-5295	DiaRD-Hpykl	24,9	N/A	N/A
**398**	Middle Ear Fluid	DNA	100	DiaRex Blood Kit	BLD-5295	DiaRD-Hpykl	23,6	N/A	N/A
**399**	Middle Ear Fluid	DNA	100	DiaRex Blood Kit	BLD-5295	DiaRD-Hpykl	26,4	N/A	N/A
**400**	Middle Ear Fluid	DNA	100	DiaRex Blood Kit	BLD-5295	DiaRD-Hpykl	29,5	N/A	N/A
